# HIV-1 Vpr—a still “enigmatic multitasker”

**DOI:** 10.3389/fmicb.2014.00127

**Published:** 2014-03-31

**Authors:** Carolin A. Guenzel, Cécile Hérate, Serge Benichou

**Affiliations:** Cochin Institute, INSERM U1016, Centre National de la Recherche Scientifique UMR8104, Université Paris-DescartesParis, France

**Keywords:** HIV-1 Vpr, reverse transcription, cell cycle, apoptosis, nuclear import

## Abstract

Like other HIV-1 auxiliary proteins, Vpr is conserved within all the human (HIV-1, HIV-2) and simian (SIV) immunodeficiency viruses. However, Vpr and homologous HIV-2, and SIV Vpx are the only viral auxiliary proteins specifically incorporated into virus particles through direct interaction with the Gag precursor, indicating that this presence in the core of the mature virions is mainly required for optimal establishment of the early steps of the virus life cycle in the newly infected cell. In spite of its small size, a plethora of effects and functions have been attributed to Vpr, including induction of cell cycle arrest and apoptosis, modulation of the fidelity of reverse transcription, nuclear import of viral DNA in macrophages and other non-dividing cells, and transcriptional modulation of viral and host cell genes. Even if some more recent studies identified a few cellular targets that HIV-1 Vpr may utilize in order to perform its different tasks, the real role and functions of Vpr during the course of natural infection are still enigmatic. In this review, we will summarize the main reported functions of HIV-1 Vpr and their significance in the context of the viral life cycle.

## Introduction

**Figure 1:**
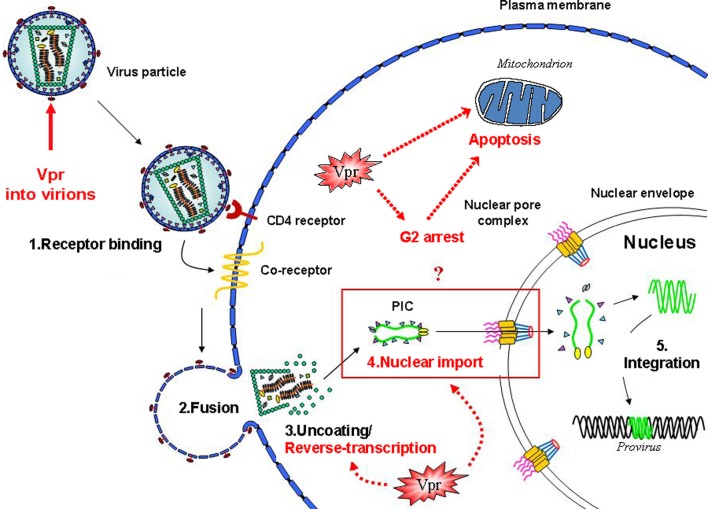
**Vpr functions and early steps of the HIV-1 life cycle**. Schematic view of the early steps of the HIV-1 infection of a target cell. The functional events in which the Vpr protein is involved are highlighted. Vpr has been shown to play multiple functions during the virus life cycle, including an effect on the accuracy of the reverse-transcription process, the nuclear import of the viral DNA as a component of the pre-integration complex, cell cycle progression, regulation of apoptosis, and the transactivation of the HIV-LTR as well as host cell genes.

The *vpr* gene is conserved among human (HIV-1 and HIV-2) and simian immunodeficiency viruses (SIV) and encodes the regulatory viral protein R (Vpr), a small basic protein (14 kDa) of 96 amino acids (Ogawa et al., 1989; Hattori et al., 1990; Steffy and Wong-Staal, 1991; Tristem et al., 1992). The importance of Vpr has been initially shown in macaque rhesus monkeys that were experimentally infected with a *vpr*-mutated SIVmac, and exhibited a decrease in virus replication and a delay in disease development progression (Lang et al., 1993; Hoch et al., 1995). *In vitro*, in the absence of Vpr, HIV-1 replicates less efficiently in macrophages, a cell type that represents an important viral reservoir by harboring the virus over long periods of time (Connor et al., 1995). Despite its small size, HIV-1 Vpr has been shown to have several roles during the viral life cycle. Due to its specific incorporation into the viral particle by interaction with the Pr55Gag-derived p6 protein, Vpr is readily present upon entry of the virus into the cell, which speaks in favor for enrollment during early steps of viral replication (see Figure 1). In this regard, Vpr has been shown to influence the reverse transcription of HIV-1 via the interaction and recruitment of the human uracil DNA glycosylase 2, an enzyme of the DNA repair machinery (Guenzel et al., 2012). A relationship that is not without controversy since different research reports argue whether UNG2 might rather have a negative impact or even no impact on HIV-1 replication (Schrofelbauer et al., 2005; Kaiser and Emerman, 2006; Yang et al., 2007). Furthermore, Vpr also affects the nuclear import of the viral DNA within the pre-integration complex (PIC), the cell cycle progression, the regulation of apoptosis and the transactivation of the HIV-1 LTR as well as host cell genes.

This review will be focused on the Vpr protein of HIV-1 and will give a summary of the multifunctional nature of Vpr during the viral life cycle in order to integrate previous and more recent studies.

## The structure of Vpr

**Figure 2:**
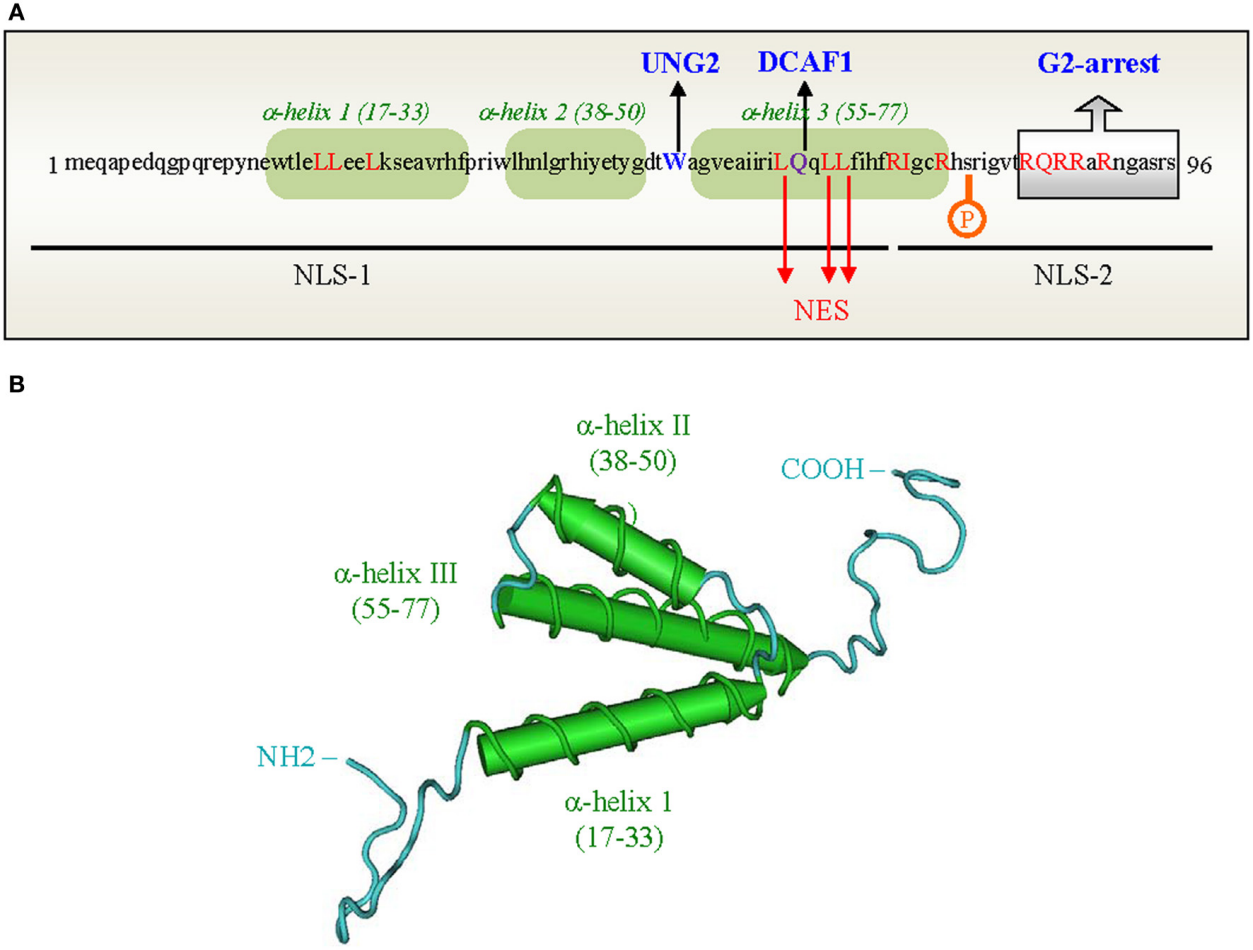
**Primary sequence and three-dimensional structure of the HIV-1 Vpr protein. (A)** Primary sequence of the Vpr protein from the HIV-1Lai strain. The 3 α-helices are boxed in green. Domains and Leu residues of Vpr involved in the nuclear import (black lines) and nuclear export (Leu in red) of proteins are indicated. The Trp residue in position 54 as well as the Gln residue critical for Vpr binding to UNG2 and DCAF1 are highlighted in blue and purple, respectively. **(B)** Three-dimensional structure of the HIV-1 Vpr protein (adapted from Morellet et al., 2003). The three α-helices (17–33, 38–50, 55–77) are colored in green, respectively; the loops and flexible domains are in blue.

HIV-1 Vpr is a relatively small protein composed of 96 amino acid residues (Figure 2A) (Checroune et al., 1995; Ramboarina et al., 2004; Kamiyama et al., 2013). The secondary and higher-order structures of Vpr have been investigated by nuclear magnetic resonance (NMR), circular dichroism (CD), and fluorescence spectroscopy (Zhao et al., 1994; Wang et al., 1996; Mahalingam et al., 1997; Kichler et al., 2000; Bruns et al., 2003; Morellet et al., 2003; Kamiyama et al., 2013). According to NMR studies on the full-length Vpr protein dissolved in acidic aqueous-organic solvents (Figure 2B) (Morellet et al., 2003), the central region of the Vpr polypeptide chain folds into three amphiphilic helices (Sherman et al., 2002; Bruns et al., 2003; Kamiyama et al., 2013). These bundled α-helices span residues 17–33, 38–50, and 55–77 and are flanked by unstructured flexible N- and C-terminal domains that are negatively or positively charged, respectively (Morellet et al., 2003). Four conserved proline residues at position 4, 10, 14, and 35 which are subjected to *cis/trans* isomerization are found in the N-terminal domain (reviewed in Bruns et al., 2003; Le Rouzic and Benichou, 2005). It was indeed reported that the cellular peptidyl-propyl isomerase cyclophilin A was able to interact with Vpr via prolines (position 14 and 35) for correct folding of the viral protein (Zander et al., 2003). The carboxy-terminal domain of Vpr contains six arginine residues between positions 73 and 96 (see Figure 2A), and this domain shows similarity to those of arginine-rich protein transduction domains. This might potentially explain the transducing properties of Vpr and its ability to cross the cell membrane lipid bilayer (Kichler et al., 2000; Sherman et al., 2002; Coeytaux et al., 2003). Additionally, the third helix of Vpr is rich in leucine residues (Schüler et al., 1999), where one side of the helix presents a stretch of hydrophobic side chains that can form a leucine-zipper like motif (Figure 2). This region was proposed to account for the formation of Vpr oligomers (Wang et al., 1996; Mahalingam et al., 1997; Schüler et al., 1999; Fritz et al., 2008) and for interaction with certain cellular partners (reviewed in Planelles and Benichou, 2009).

Vpr has been shown to exist as dimers, trimers, tetramers and higher order multimers (Zhao et al., 1994), however it is still not completely elucidated how the dimeric or multimeric states of the protein affect the different functions of Vpr. A real-time study using a flow cytometry fluorescence resonance energy transfer has confirmed that Vpr self-associates within live cells (Bolton and Lenardo, 2007). Self-association was dependent on the hydrophobic patch that is located on the third α-helix and mutations in this region did not impair the ability of Vpr to induce G2 arrest, suggesting that oligomerization of Vpr is not absolutely required for the functions of the protein. In addition, mutations in the arginine-rich domain, such as R80A and R87/88A, did not impair self-association but were unable to induce G2 arrest (Bolton and Lenardo, 2007). Therefore, it appears that Vpr does not require oligomerization toward induction of the cell cycle blockage but the exposed hydrophobic amino acids in the amino-terminal helix-1 are important for the cell cycle arrest and cytopathogenic functions of Vpr (Barnitz et al., 2011). A more recent study reports that oligomerization of Vpr is essential for incorporation into virus particles (Venkatachari et al., 2010). Moreover, it has been recently proposed that Vpr may assume an antiparallel helical dimer with the third α-helices of the two subunits facing each other, and the His71 and Trp54 play a crucial role in this dimer formation (Kamiyama et al., 2013).

## Vpr is incorporated into virus particles

Vpr is expressed at a late stage of the virus life cycle, but it is present during the early steps of infection in target cells since it is packaged into virions that were released from the producing cells. The incorporation of Vpr occurs through a direct interaction with the carboxy-terminal p6 region of the *gag*-encoded Pr55Gag precursor (Bachand et al., 1999; Paillart and Göttlinger, 1999; Selig et al., 1999; Jenkins et al., 2001a,b). The integrity of the α-helices of Vpr is required for efficient packaging into virions (Singh et al., 2000), and a leucine-rich (LR) motif found in the p6 region of the Pr55Gag precursor is directly involved in the interaction with Vpr (Kondo and Göttlinger, 1996; Selig et al., 1999; Jenkins et al., 2001a,b; Fritz et al., 2010). The Pr55Gag p6 region has also been found to be phosphorylated during HIV-1 infection by atypical protein kinase C (Hemonnot et al., 2004) regulating the incorporation of Vpr into HIV-1 virions and thereby supporting virus infectivity (Kudoh et al., 2014). After assembly and proteolytic cleavage of Pr55Gag in matrix, capsid, nucleocapsid (NCp7), and p6 mature proteins, Vpr is recruited into the conical core of the virus particle (Accola et al., 2000; Welker et al., 2000) where it is tightly associated with the viral RNA (Zhang et al., 1998; De Rocquigny et al., 2000). Interestingly, Vpr displays a higher avidity for NCp7 than for the mature p6 protein (Dong et al., 1997; Selig et al., 1999; Jenkins et al., 2001a,b). Since p6 is excluded from the virion core (Accola et al., 2000; Welker et al., 2000), Vpr could switch from the p6Gag region of the precursor to the mature NCp7 protein in order to gain access to the core of the infectious virus particle budding at the cell surface. In any case, p6 has been reported to show a high affinity for membrane bilayers which substantially increases the interaction between p6 and Vpr (Salgado et al., 2009). It was estimated that Vpr is efficiently incorporated in HIV-1 virions with a Vpr/Pr55Gag ratio of ~1:7 (Knight et al., 1987; Cohen et al., 1990; Welker et al., 2000), which may represent 275 molecules of Vpr per virion. More recently it has been shown that the HIV-1 Pr55Gag precursor induces the recruitment of Vpr oligomers to the plasma membrane (Fritz et al., 2010). Vpr oligomerization has been found to be essential for binding of Vpr to Pr55Gag and for its accumulation at the plasma membrane early during Pr55Gag assembly, but the exact role of these oligomers is not certain yet (Fritz et al., 2010).

The incorporation of Vpr has also been used as a unique tool to target cargoes such as cellular and viral proteins or drugs into viral particles (Wu et al., 1995; Yao et al., 1999; Fritz et al., 2010). This property found extensive use in studies that evaluated the respective functions of integrase (IN) and reverse transcriptase (RT) during virus replication by expressing Vpr-IN and Vpr-RT fusions in *trans* in virus-producing cells (Wu et al., 1997, 1999; Liu et al., 1999). Furthermore, Vpr fused to the green fluorescence protein (GFP) has been used to tag HIV particles in order to follow intracellular virus behavior during the early intracellular steps of infection in target cells (Loeb et al., 2002; Steffens and Hope, 2003; Fritz et al., 2010).

## Vpr and the cell cycle

Among the range of functions of the Vpr protein, the Vpr-dependent G2 arrest activity was extensively explored since it was described for the first time in 1995 (He et al., 1995; Jowett et al., 1995; Re et al., 1995; Rogel et al., 1995). The Vpr protein encapsided into HIV-1 virions is able to block proliferation of newly infected T lymphocytes. Following infection, these cells accumulate at the G2-M phase and show a 4N DNA content. The first studies proposed that the presence of Vpr leads to the accumulation of the hyperphosphorylated form of the cyclin-dependent kinase CDC2 (the complex p34 cdc2/cyclin B). This inactive form of the complex would be able to block the cell cycle before the mitosis.

This cytostatic function of Vpr is well conserved among primate lentiviruses (Planelles et al., 1996; Stivahtis et al., 1997), and could be a strategy used by HIV and SIV to improve viral replication and protein expression, and even to reactivate the virus through an epigenetic control of the LTR promoter (Yao et al., 1998; Thierry et al., 2004). The biological significance of this cell cycle arrest during the natural infection is not well understood, but the HIV-1 LTR seems to be more active in the G2 phase, implying that the G2 arrest may confer a favorable cellular environment for efficient transcription of HIV-1 (Goh et al., 1998). In agreement, the Vpr-induced G2 arrest correlates with high level of viral replication in primary human T cells. Overexpression of dominant negative mutant of the p34 cdc2 kinase shows that Vpr-induced G2 arrest correlates with HIV-1 activation (Goh et al., 1998). Vpr might also be involved in virus activation through other interactions such as the formation of a complex with p53 and the transcription factors Sp1 (Wang et al., 1995; Sawaya et al., 1998). This complex could lead to the activation of the p21/WAF promoter resulting in the transactivation of the viral LTR (Cui et al., 2006). Using a human hematopoietic stem cell-transplanted humanized mouse model, it was recently shown that Vpr causes G2 cell cycle arrest and apoptosis predominantly in proliferating CCR5+ CD4+ T cells, which mainly consist of regulatory CD4+ T cells (Tregs), resulting in Treg depletion and enhanced virus production during acute infection *in vivo* (Sato et al., 2013). In addition, recent results just published by Laguette et al. (2014) show that the interaction of Vpr with the structure-specific endonuclease (SSE) regulator SLX4 complex (SLX4com) is crucial for the G2-arrest activity but also for escape of HIV-1 from innate immune sensing in infected cells.

Some studies try to correlate the Vpr structure with cell cycle regulation. Historically, this function of Vpr was associated with the helix-3 and the flexible C-terminal part of the protein (Marzio et al., 1995; Mahalingam et al., 1997; Chen et al., 1999). Some key phosphorylations of the C-terminus part have also been associated with the G2 arrest, such as phosphorylation of the Ser79 residue (see Figure 2A) (Zhou and Ratner, 2000). Vpr is mainly localized in the nucleus and at the nuclear envelope where previous reports indicated it could induce herniations and burstings of the nuclear membrane and even defects in the nuclear lamina (de Noronha et al., 2001; Sörgel et al., 2012). These morphological modifications could impact several nuclear factors and redistribute a large range of proteins from the nucleus to the cytoplasm leading to alteration of the cell cycle. Indeed, the cyclins involved in the cell cycle regulation are closely regulated and their spatio-temporal distribution is primordial for the continuity of the cell cycle. More recently, interactions between Vpr and chromatin have been reported (Belzile et al., 2010; Shimura et al., 2011). Vpr can cause epigenetic disruption of heterochromatin by inducing the displacement of heterochromatin protein 1-α (HP1-α) through acetylation of the histone H3 and causes premature chromatids separation and consequently G2 arrest (Shimura et al., 2011). The interaction between Vpr and the chromatin should target and activate the ataxia telangiectasia mutated and Rad3-related kinases ATM/ATR, two of the main sensors of the cell cycle (Koundrioukoff et al., 2004). The link between ATR and the Vpr-dependent G2 arrest was initially reported by Roshal et al. (Roshal et al., 2003) (for review on ATR pathway, see Sørensen and Syljuåsen, 2012). The ATR and ATM proteins control the G2 arrest provoked by DNA damage but it is controversial if Vpr really causes DNA damage or just mimics this damage and activates some sensors involved in this process (Cliby et al., 2002). It was reported that the inhibition of ATR abrogates the Vpr-dependent G2 arrest. Following ATR activation by Vpr, Chk1 is activated through phosphorylation and required for the G2 arrest (Li et al., 2010). Clearly, Vpr acts on the cell cycle by a cascade of reversible phosphorylations. The expression of Vpr correlates with inactivation of the p34/cdc2 CDK1 kinase associated with cyclin B. Cdc2 is normally activated by the cdc5 phosphatase which is inactive in its hypophosphorylated form in Vpr-expressing cells (He et al., 1995; Re et al., 1995), whereas Wee1 inhibits the cdc2 kinases (Sørensen and Syljuåsen, 2012). Vpr seems to be able to directly activate the Wee1 protein by binding to its “N” lobe but this interaction is not sufficient for induction of the G2 arrest (Kamata et al., 2008). However, other key regulators of the cell cycle interacting with Vpr could be members of the 14-3-3 protein family (Kino et al., 2005) which bind phosphorylated serine/threonine proteins such as the cell cycle regulators Wee1, Cdc25, and Chk1. Consequently, 14-3-3 could regulate activities and distribution of these proteins (Lopez-Girona et al., 1999; Hermeking and Benzinger, 2006). These authors revealed that overexpression of 14-3-3 leads to an increase of the cell cycle arrest in the presence of Vpr while the absence of this scaffolding protein reduces the Vpr-induced activity. Another study revealed how Vpr disrupts 14-3-3θ from centrosome and increases its association with the importin β, Cyclin B1, and Cdk1 (Bolton et al., 2008).

Today, almost all the new studies about the Vpr-induced G2 arrest try to identify the potential target of Vpr degraded by the proteasome machinery. Indeed, several groups clearly showed that Vpr connects the DCAF1 adaptor of the Cul4A ubiquitin ligase to a so far unidentified host target protein linked to the G2 arrest (Belzile et al., 2007; DeHart et al., 2007; Le Rouzic et al., 2007; Schrofelbauer et al., 2007). First, the interactions between Vpr and cullins 1 and 4 (Cul1, Cul4), belonging to the ubiquitin ligase complex, were reported (Schrofelbauer et al., 2007). Then, the Vpr-binding protein (VprBP) was described as a substrate specificity module in Cul4 and DDB1 (damaged-DNA specific binding protein 1)-based ubiquitine ligase E3 complexes (Angers et al., 2006; He et al., 2006; Higa et al., 2006a; Jin et al., 2006). Furthermore, other teams described a larger complex where Vpr was associated with Cul4A, DDB1, Rbx2/Roc1 and an ubiquitin-conjugating enzyme or E2. At the same time VprBP was renamed DDB1-and Cul4-associated factor (DCAF)-1 (Belzile et al., 2007; DeHart et al., 2007; Hrecka et al., 2007; Le Rouzic et al., 2007; Schrofelbauer et al., 2007; Tan et al., 2007; Wen et al., 2007). The Cul4-DDB1-E3 ligase complex can bind several DCAFs and seems involved in the maintenance and control of the genome stability, DNA replication and cell cycle check point control (Sugasawa et al., 2005; Higa et al., 2006b; Wang et al., 2006). From these studies, a model where Vpr binds the Cul4-DDB1-DCAF1 E3 ligase to trigger the degradation of a putative protein responsible for the G2 arrest has emerged (Dehart and Planelles, 2008). In this model, Vpr uses two distinct interfaces for binding, one for the attachment to VprBP/DCAF1 and the other for the putative substrate protein. Vpr binds DCAF1 through the LR motif found between amino acids 60 and 68 while the C-terminal basic flexible region binds to the substrate to be ubiquitinylated and degraded and responsible for G2 arrest (Zhao et al., 1994; DeHart et al., 2007; Le Rouzic et al., 2007). Recently, Belzile et al. (2010) proposed that Vpr is present in the nucleus and more specifically inside nuclear foci where it is associated with VprBP and the DDB1-CUL4A-E3 ubiquitine ligase. These foci colocalize with DNA repair foci containing proteins such as γH2AX and RPA2. This association may lead to the recruitment and the degradation of a chromatin-bound substrate via a K48-linked polyubiquitinylation (Belzile et al., 2010) which activates the key protein ATR and the G2 arrest. Finally, a new essential actor of the Vpr-dependent G2 arrest, the SSE regulator SLX4com has been recently identified by proteomic analysis (Laguette et al., 2014). Vpr activates SLX4com through direct interaction with SLX4 leading to the recruitment of VprBP and the kinase-active PLK1. This association would lead to the cleavage of DNA by SLX4-associated MUS81-EME1 endonucleases. Vpr activation of premature MUS81-EME1 induces accumulation of FANCD2 foci and consequently DNA intermediates cleavage and replication stress.

## Vpr and apoptosis

Acute phase of AIDS is characterized by a net decrease of CD4+ T cells, and the hallmark of the chronic phase is a gradual decrease of the peripheral CD4+ T cells. While the virus mainly targets lymphocytes and macrophages, no depletion of macrophages has been reported and these terminally-differentiated cells may rather serve as virus reservoirs. The reason why infected macrophages were not susceptible to apoptosis has been recently explored. Using macrophage-like cells derived from differentiated THP1 CD4+ myeloid cells, a recent report showed that Vpr is not able to downregulate the anti-apoptotic protein cIAP1/2 (Busca et al., 2012). However, Mishra et al. (2007) previously revealed the possibility that the C-terminal part of Vpr could induce apoptosis in monocytes via a JNK-dependant pathway.

Although different HIV-1-induced pathways for apoptosis induction have been described, Vpr appears as one of the main actors of the cell death observed during HIV-1 infection. However, it is still controversial how Vpr induces apoptosis and/or necrosis. Moreover, uninfected bystander T cells can be also targeted by Vpr, since Vpr can get access to the extracellular compartment like a soluble protein (Reiss et al., 1990; Cummins and Badley, 2010; Abbas, 2013). A previous model for Vpr-induced apoptosis proposed that Vpr would be able to bind the WxxF motif of the transmembrane adenine nucleotide transporter (ANT) protein exposed in the inner membrane of mitochondria. Jacotot et al. (2000, 2001) were the first to detect this interaction and found that Vpr could also bind to another member of the permeability transition pore complex (PTPC), the voltage-dependent anion channel (VDAC). This team showed the capacity of a synthetic Vpr polypeptide to trigger permeabilization of the mitochondrial membrane resulting in the collapse of the mitochondrial transmembrane potential. Following permeabilization of both inner and outer mitochondrial membranes (Ghiotto et al., 2010), the release of pro-apoptotic proteins like the cytochrome c forms the apoptosome with the caspase 9 and Apaf-1 and allows recruitment of caspase 3. Bax, another pore forming complex protein should also be involved in the Vpr-induced cell death since a conformational change and activation of Bax was detected in apoptotic cells expressing Vpr (Andersen et al., 2006). In this study, the authors characterized cell death in mice, and described that ANT may promote a necrotic cell death rather than apoptosis.

It was indeed discussed whether the Vpr-induced G2 arrest was linked to the observed apoptosis in Vpr expressing cells. Earlier, some studies concluded that Vpr-induced apoptosis was independent of the G2 arrest activity (Nishizawa et al., 2000a,b) showing that a C-terminal truncated form of Vpr still induced apoptosis but did not induce G2 arrest. However, others and more recent studies found a correlation between both Vpr activities and suggested that apoptosis was a consequence of the prolonged G2 arrest (Andersen et al., 2006). According to Stewart and colleagues, apoptosis would happen in cells after the G2 arrest as a consequence of the blockage, and this was observed in human fibroblasts, T cell lines, as well as primary peripheral blood lymphocytes (Stewart et al., 1997, 1999). Accordingly, Zhu et al. (2001) showed that treating cells with caffeine, an inhibitor of both ATM and ATR, which are key proteins involved in cell cycle control, abrogated both G2 arrest and apoptosis. Trying to understand this ATR-dependent mechanism, subsequent studies from the same team described an activation of the DNA repair enzyme BRCA1 leading to the regulation of the growth arrest and DNA damage protein 45α (GADD45α) involved in the cell death process (Zimmerman et al., 2004; Andersen et al., 2005). Moreover, some cell cycle regulators such as Wee1 and Chk1 could also be involved in the Vpr-dependent apoptosis pathway. The Vpr-dependent phosphorylation of Chk1, an event that begins during S phase of the cell cycle, could also trigger apoptosis (Li et al., 2010).

Furthermore, it was reported that Vpr may impact the immune system homeostasis by stimulating the secretion of TNF-α by dendritic cells, resulting in apoptosis of CD8+ T cells (Majumder et al., 2007). Vpr could also increase expression of the NKG2D stress ligand in CD4+ T cells promoting their destruction by the Natural Killer (NK) cells (Ward et al., 2009; Richard et al., 2010). According to Ward et al. (2009), this mechanism causes a link between the G2 arrest and the apoptosis since they showed NKG2D expression is dependent of ATR activation by Vpr.

Finally, some studies revealed that 40–50% of HIV-1 seropositive patients have neurocognitive disorders (Ances and Ellis, 2007; Jones et al., 2007; Harezlak et al., 2011), and different theories have been proposed to explain these neurological disorders. Among the Vpr effects, some hypothesized that extracellular Vpr might be able to enter into neurons (Rom et al., 2009) where it can cause apoptosis. Jones et al. (2007) tested the effect of soluble Vpr in neurons and detected apoptosis involving cytochrome c release, p53 induction, and activation of caspase-9. This study also suggested that Vpr triggers the release of the inflammatory IL-6 cytokine by astrocytes which could affect neuron survival. More recently, it was shown that Vpr could also act on the glycolytic pathway of astrocytes leading to secretion of stress molecules (Ferrucci et al., 2013).

## Vpr and the reverse transcription

After virus entry, the viral core is released into the cytoplasm of the target cell where the reverse transcription of the viral RNA takes place within a large nucleoprotein complex (Farnet and Haseltine, 1991; Bukrinsky et al., 1993; Miller et al., 1997; Fassati and Goff, 2001; Nermut and Fassati, 2003; Lyonnais et al., 2013). This reverse transcription complex (RTC) contains the two copies of viral RNA and the viral RT, IN, NCp7, Vpr and a few molecules of the matrix protein. It is generally believed that the reverse transcription process is initiated in virus particles and then completed in the cytoplasm after the virus has entered into the target cell (Figure 1). The reverse transcription process is likely to take place in parallel during both virus uncoating and trafficking through the cytoplasm (for reviews, see Goff, 2001; and Pomerantz, 2000). Several studies confirmed that Vpr co-localizes with the viral nucleic acids and IN within purified HIV-1 RTCs (Fassati et al., 2003; Nermut and Fassati, 2003; Steffens and Hope, 2003), and remains associated with the viral DNA within 4–16 h after infection (Fassati and Goff, 2001). Interestingly, Vpr has recently been reported to be essential for unintegrated HIV-1 gene expression and *de-novo* virus production in a virus replication pathway utilizing RT DNA products that failed to integrate (Poon and Chen, 2003; Trinité et al., 2013).

In addition to a potential role in the initiation step of the reverse transcription process (Stark and Hay, 1998), it has been shown that Vpr modulated the *in vivo* mutation rate of HIV-1 by influencing the accuracy of the reverse transcription. The HIV-1 RT is an error-prone RNA-dependent DNA polymerase, and quantification of the *in vivo* rate of forward virus mutations per replication cycle revealed that the mutation rate was 4-fold higher in the absence of Vpr expression when measured in dividing cells using a genetically engineered system (Mansky and Yemin, 1995; Mansky, 1996). Furthermore, analysis in non-dividing cells showed that this phenotype is even more pronounced in primary monocyte-derived macrophages (MDMs) leading to a 16-fold increase of the HIV-1 mutation frequency (Chen et al., 2004). Strikingly, this activity correlates with the interaction of Vpr with the nuclear form of uracil DNA glycosylase (UNG2) (Mansky et al., 2000), an enzyme of the base excision repair system that specifically removes the RNA base uracil from DNA. The inclusion of uracil in DNA can occur either by misincorporation of dUTP or by cytosine deamination. While the Trp residue in position 54 located in the exposed loop connecting the second and the third α-helix of HIV-1 Vpr has been shown to be critical to maintain the interaction with UNG2, the Vpr-binding site was mapped within the C-terminal part of UNG2, and occurs through a WxxF motif. So far, three distinct cellular partners of Vpr were known to contain a WxxF motif: the TFIIB transcription factor, the adenosine-nucleotide translocator (ANT) and UNG2 (as reviewed in Planelles and Benichou, 2009). However, the WxxF motif is not sufficient for Vpr binding, since other cellular Vpr-interacting proteins, such as DCAF1 or DICER for example, still bind to Vpr independently of the presence of a WxxF motif within their primary sequence (Belzile et al., 2007; DeHart et al., 2007; Le Rouzic et al., 2007; Schrofelbauer et al., 2007; Casey Klockow et al., 2013).

Some authors suggested that the association of Vpr with UNG2 in virus-producing cells allows the incorporation of a catalytically active enzyme into HIV-1 particles where UNG2 may directly influence the reverse transcription accuracy (Mansky et al., 2000; Chen et al., 2004), and this plays a specific role in the modulation of the virus mutation rate. The model supporting the direct contribution of incorporated UNG2 in the reverse transcription process was demonstrated by using an experimental system in which UNG2 was recruited into virions independently of Vpr. UNG2 was expressed as a chimeric protein fused to the C-terminal extremity of the VprW54R mutant, a Vpr variant that fails to recruit UNG2 into virions and to influence the virus mutation rate, even though it is incorporated as efficiently as the wild-type Vpr protein. The VprW54R-UNG2 fusion is also efficiently packaged into HIV-1 virions and can restore a mutation rate equivalent to that observed with wild-type Vpr, both in actively dividing cells and in MDMs. In agreement with this phenotype on the virus mutation frequency, it was finally documented that the Vpr-mediated incorporation of UNG2 into virus particles contributed to the ability of HIV-1 to replicate in primary macrophages. When the VprW54R variant was introduced into an infectious HIV-1 molecular clone, virus replication in macrophages was both reduced and delayed. Although it was proposed that the viral integrase was also able to mediate interaction with UNG2 (Priet et al., 2003), Vpr seems to be the main viral determinant that allows for the incorporation of UNG2 into virus particles. However, further analyses are required to document the nature of interactions between UNG2, Vpr, IN as well as RT both in virus-producing cells and in target cells.

Other studies also confirmed that UNG2 was efficiently recruited into virus particles (Priet et al., 2005; Kaiser and Emerman, 2006; Yang et al., 2007; Jones et al., 2010), indicating that this recruitment might influence the accuracy of the reverse transcription process and has a positive influence on viral replication (Chen et al., 2004; Priet et al., 2005; Jones et al., 2010). Interestingly, it has been recently reported that HIV-1 DNA generated in infected macrophages and CD4-positive T cells is heavily uracilated (Yan et al., 2011). However, the specific role of UNG2 incorporation into virions was challenged by other studies (Schrofelbauer et al., 2005; Kaiser and Emerman, 2006; Yang et al., 2007). While the specificity of the interaction between Vpr and UNG2 was not questioned, these studies suggested a detrimental (Schrofelbauer et al., 2005; Yang et al., 2007; Eldin et al., 2013) or dispensable (Kaiser and Emerman, 2006) effect of UNG2 on virus replication. In the model suggesting a detrimental effect on UNG2 on virus replication, Vpr was shown to induce the proteasomal degradation of UNG2 in virus-producing cells in order to prevent its recruitment into virus particles (Schrofelbauer et al., 2005, 2007; Eldin et al., 2013). It has also been reported that the Vpr-UNG2 interaction temporarily impairs the uracil excision activity of UNG2 in infected cells (Eldin et al., 2013). However, other data have indicated that the Vpr-induced reduction of endogenous UNG2 observed in HIV-1 infected cells was not solely related to proteasomal degradation (Langevin et al., 2009; Nekorchuk et al., 2013) and that UNG2 might not be responsible for the degradation of HIV-1 DNA containing misincorporated dUTP which prevents viral integration (Weil et al., 2013). More recently, it has been argued that incorporation of UNG2 into HIV-1 particles may not be detrimental for virus infection in target cells but rather has a positive impact on virus replication and virus infectivity achieved through a non-enzymatic mechanism mapping within a 60-amino-acid long domain located in the N-terminal region of UNG2 (Guenzel et al., 2012). Interestingly, this domain is also required for interaction of UNG2 with the p32 subunit (RPA2) of the replication protein A complex (Nagelhus et al., 1997; Otterlei et al., 1999; Mer et al., 2000; De Silva and Moss, 2008). It was observed that enforced virion recruitment of UNG2, through UNG2 overexpression in virus producing cells, similarly influenced infectivity of X4 and R5 HIV-1 strains in transformed cell lines and MDMs, respectively (Guenzel et al., 2012), which stands in contrast to another report suggesting that UNG2 was exclusively required for efficient infection of primary cells by R5-tropic viruses (Jones et al., 2010). Strikingly, viruses produced from cells depleted of endogenous UNG2 and RPA2 resulted in significantly reduced infectivity and replication, the latter evidenced by a reduced amount of viral transcripts measured during the reverse transcription process (Guenzel et al., 2012). These new intriguing findings are not yet completely understood and further investigations are needed to clarify the mechanism.

HIV-1 and other lentiviruses are unusual among retroviruses in their ability to infect resting or terminally differentiated cells. While Vpr has been shown to facilitate the nuclear import of viral DNA in non-dividing cells (see below), the virion incorporation of UNG2 via Vpr may also contribute to the ability of HIV-1 to replicate in primary macrophages. This implies that UNG2 is a cellular factor that plays an important role in the early steps of the HIV-1 replication cycle (i.e., viral DNA synthesis). This observation is in agreement with a report showing that the misincorporation of uracil into minus strand viral DNA affects the initiation of the plus strand DNA synthesis *in vitro* (Klarmann et al., 2003). This observation suggests that UNG2 is likely to be recruited into HIV-1 particles to subsequently minimize the detrimental accumulation of uracil into the newly synthesized proviral DNA. While further works are needed to explain the precise mechanism for how the UNG2 catalytic activity may specifically influence HIV-1 replication in macrophages, it is worth noting that non-dividing cells express low levels of UNG and contain relatively high levels of dUTP (Chen et al., 2002). Similarly, most non-primate lentiviruses, such as feline immunodeficiency virus (FIV), caprine-arthritis-encephalitis virus (CAEV) and equine infectious anemia virus (EIAV), have also developed an efficient strategy to reduce accumulation of uracil into viral DNA. These lentiviruses encode and package a viral-encoded dUTP pyropshophatase (dUTPase) into virus particles, an enzyme that hydrolyzed dUTP to dUMP, and thus maintains a low level of dUTP. Interestingly, replication of FIV, CAEV, or EIAV that lack functional dUTPase activity is severely affected in non-dividing host cells (e.g., primary macrophages). Taken together, these results indicate that uracil misincorporation in viral DNA strands during reverse transcription is deleterious for the ongoing steps of the virus life cycle. The presence of a viral dUTPase or a cellular UNG will prevent these detrimental effects for replication of non-primate and primate lentiviruses in macrophages, respectively.

Finally, it is intriguing to note that two viral auxiliary proteins from HIV-1, Vpr and Vif, can both influence the fidelity of viral DNA synthesis. The Vif protein forms a complex with the cellular deaminase APOBEC3G thereby preventing its encapsidation into virions (Sheehy et al., 2002; Lecossier et al., 2003; Mangeat et al., 2003; Mariani et al., 2003; Stopak et al., 2003), while Vpr binds the DNA repair enzyme UNG2. In this context it was suggested that incorporation of UNG2 into viral particles would have a detrimental effect on reverse transcription by introducing a basic sites into viral DNA in regards to uracil residues resulting from cytosine deamination by the cytidine deaminase APOBEC3G (Schrofelbauer et al., 2005; Yang et al., 2007). While the specific role of UNG2 in the antiviral activity of APOBEC3G was not directly questioned (Schrofelbauer et al., 2005), others reported data indicating that the antiviral activity of overexpressed APOBEC3G was partially affected when viruses were produced in UNG2-depleted 293T cells (Yang et al., 2007). However, these data are in apparent contradiction with results from other reports in which viruses were produced in UNG2-depleted cells which expressed or did not express APOBEC3G (Priet et al., 2005; Jones et al., 2010; Guenzel et al., 2012), but also from reports showing that APOBEC3G-mediated restriction of HIV-1 was independent of UNG2 (Kaiser and Emerman, 2006; Langlois and Neuberger, 2008). More recently, and in favor for a correlative positive impact of UNG2, it has been shown that the detrimental hypermutation of Hepatitis B virus DNA induced by either APOBEC3G or interferon treatment was enhanced in a human hepatocyte cell lines when UNG2 activity was inhibited (Kitamura et al., 2013; Liang et al., 2013). Additional investigations are thus required to further understand this apparent contradiction regarding the role of UNG2 for the antiviral activity of APOBEC restriction factors. However, it is tempting to speculate that the action of both viral proteins may influence the mutation rate during the course of HIV-1 infection, and their balance may play a key role during disease progression and antiretroviral treatment susceptibility in infected individuals (Fourati et al., 2010, 2012).

## Vpr and the viral DNA nuclear import

Like other retroviruses, HIV-1 has the capacity to infect and integrate its genomic DNA into dividing cells like T lymphocytes, but lentiviruses are also remarkable by their capacity to infect non-dividing cells, in contrast to onco-retroviruses which need the disintegration of the nuclear envelope to allow access of their genome for integration in the host genome (Greber and Fassati, 2003). Indeed, HIV-1 can infect terminally-differentiated macrophages and produces new virions after integration of its DNA into the cell genome. The Vpr protein has been described as a potential enhancer of HIV-1 replication especially in macrophages whereas it does not impact on virus replication in proliferating T cells (Balliet et al., 1994; Connor et al., 1995; Eckstein et al., 2001). In macrophages, the viral DNA needs to be transported into the interphasic nucleus by an active mechanism (Vodicka et al., 1998). After virus entry into the cell, the viral genome is reverse-transcribed in full length viral double-strand DNA which is associated with viral and host cell proteins into the so-called PIC. Among the protein components of the PIC, four viral proteins have been detected (e.g., the reverse-transcriptase and integrase enzymes, the matrix protein and Vpr) (Heinzinger et al., 1994; Jenkins et al., 1998; Eckstein et al., 2001; Le Rouzic et al., 2002; Schang, 2003; Suzuki et al., 2009).

Despite the absence of a basic canonical or a M9-dependant nuclear localization signal (NLS) in the protein sequence, Vpr shows evident karyophilic properties (Gallay et al., 1996; Jenkins et al., 1998; Depienne et al., 2000). Finally, Vpr seems to use a non-classical pathway to be transferred in the nucleus through direct binding to importin-α (Gallay et al., 1996; Nitahara-Kasahara et al., 2007). However, it was largely shown that Vpr is able to shuttle between the cytoplasm and the nuclear compartments and could play a potential role in transport of the viral DNA (Jenkins et al., 2001a,b; Sherman et al., 2001, 2003; Le Rouzic et al., 2002). By using a photobleaching strategy on living cells, Le Rouzic and colleagues revealed that Vpr-GFP has shuttling properties (Le Rouzic et al., 2002). This activity was linked to the distal LR region, a classical nuclear export signal (NES) recognized by the CRM1-dependent pathway (Sherman et al., 2001, 2003). This NES could be involved in the release of Vpr back into the cytoplasm, making it available for virion packaging through interaction with the Pr55Gag precursor (Jenkins et al., 2001a,b).

The role of Vpr within the PIC has been studied in living cells through tracking of a GFP-tagged form of Vpr (McDonald et al., 2002). These authors evidenced a tight association between the PIC and the cytoplasmic microtubules, targeting the viral DNA toward the nucleus. The PIC moves along the cytoskeletal microtubule filaments using the dynein/dynactin complex as a motor, leading to its accumulation in the perinuclear region close to the centrosome. So far, it is not known if Vpr plays an active role in the intracytoplasmic transport of the PIC; it may be only associated with the complex and play a role later for nuclear membrane anchoring and translocation of the viral DNA into the nucleus (for review, Le Rouzic and Benichou, 2005).

The nuclear envelope contains two concentric membranes with nuclear pore complexes (NPC) consisting of aqueous channels which allow for selective transport between the cytoplasmic and nuclear compartments. The NPC corresponds to a 125 MDa structure consisting of 30 distinct nuclear pore proteins, named nucleoporins (Nups) (Cronshaw et al., 2002). Most of these Nups have filamentous structures containing FG or FxFG motif repeats emanating from both sides of the NPC and able to dock transport factors (Rout and Aitchison, 2001). It was initially reported that Vpr was able to recognize these FG motifs in Nups such as p54 and p58 leading to the docking of Vpr to the nuclear membrane (Fouchier et al., 1998; Popov et al., 1998). Another interaction with the human CG1 (hCG1) nucleoporin has been described by Le Rouzic et al. (2002). However, Vpr associated with the N-terminal region of hCG1 while the FG repeats of this Nup were located in the C-terminal part of the protein. This interaction results in Vpr accumulation at the nuclear envelope, which is believed to be involved in active nuclear import of the PIC in non-dividing cells, such as macrophages (Jacquot et al., 2007). Through these interactions with components of NPC, Vpr may be responsible for the first step of viral DNA import by targeting the PIC to the nuclear pore complex while other components of the PIC could trigger the next step of the nuclear translocation. As mentioned above, it was also reported that Vpr can induce herniations and the dissociation of lamina and nuclear envelope which provoke a blend of nuclear and cytoplasmic proteins (de Noronha et al., 2001). The exact mechanism inducing these membrane perturbations is not understood, but some authors hypothesize that the interaction of Vpr with the NPC proteins could impact nuclear membrane stability. Consequently it may also facilitate the entry of the PIC through a non-conventional pathway (Segura-Totten and Wilson, 2001).

## Conclusions and future directions

Like other HIV-1 auxiliary proteins, Vpr is a small but multifunctional protein which is potentially able to interact with plenty of cellular partners. During the last two decades, several groups looked for such partners but the importance of such interactions often needs to be better documented to support their real impact on HIV-1 propagation, immune and antiretroviral treatment evasion and disease progression. While major efforts have been made during the last years to better define the molecular mechanisms and cellular targets of Vpr, additional works are needed for the complete understanding of its wide range of activities in key processes during the early steps of the viral life cycle (i.e., reverse transcription, intra-cytoplasmic routing and nuclear import of the viral DNA). However, precise characterization of Vpr interactions leading to the proteasomal degradation of some host cell factors is certainly the main challenge for a better understanding of the Vpr contribution to the overall pathogenesis of HIV-1 infection.

### Conflict of interest statement

The authors declare that the research was conducted in the absence of any commercial or financial relationships that could be construed as a potential conflict of interest.
